# Tuning the Functionality by Splicing: Factor H and Its Alternative Splice Variant FHL-1 Share a Gene but Not All Functions

**DOI:** 10.3389/fimmu.2020.596415

**Published:** 2020-10-15

**Authors:** Marco Mannes, Arthur Dopler, Markus Huber-Lang, Christoph Q. Schmidt

**Affiliations:** ^1^ Institute of Clinical and Experimental Trauma Immunology, University Hospital of Ulm, Ulm, Germany; ^2^ Institute of Pharmacology of Natural Products and Clinical Pharmacology, Ulm University, Ulm, Germany

**Keywords:** complement system, factor H, Factor H-like protein 1, cell protection, regulatory selectivity

## Abstract

The alternative pathway regulator Factor H-like protein 1 (FHL-1) is composed of the first 7 N-terminal complement control protein domains of Factor H (FH) and protects host surfaces from uncontrolled complement attack. Although FHL-1 shares the N-terminal regulatory domains with FH, it was thought to be a weaker regulator. Recently, the regulatory activity of FHL-1 was shown to be comparable to FH. Nonetheless, the question remained whether FHL-1 is an indispensable, unique regulator. The discovery that FHL-1 is the predominant regulator on Bruch’s membrane, a critical site for the onset and progression of age-related-macular degeneration (AMD), showed that FHL-1 is essential for complement regulation. A common single nucleotide polymorphism in FH/FHL-1 that predisposes for AMD underlines the important role of FHL-1 in this context. Reports that some cancer tissues specifically upregulate FHL-1 expression, thereby evading immune surveillance, suggests a pronounced regulatory activity of the splice variant. Several microorganisms specifically recruit FHL-1 to evade complement attack. From a phylogenetic point of view, FHL-1 appears much later than other complement regulators, which could imply a specific role that is possibly not systemic but rather tissue specific. This review focuses on the current knowledge of FHL-1 and its physiological and pathophysiological roles.

## Introduction

The complement system is an essential part of the innate immune system protecting against infections and helping in maintaining tissue homeostasis. While the classical and lectin pathways are activated specifically, the activation of the alternative pathway (AP) occurs indiscriminately by spontaneous hydrolysis of C3, yielding C3(H_2_O) (1). C3b produced by any initiating pathway in turn becomes amplified further by the AP amplification loop. This indiscriminate generation and surface deposition of C3b necessitates precise regulation to specifically downregulate AP amplification on self-surfaces. Therefore, only foreign, dangerous or impaired host surfaces allow unlimited or under-regulated AP activity. To protect themselves from AP-mediated attack, human cells and surfaces are equipped with preformed regulators of defense, that is, complement regulatory proteins (2). These are membrane-bound regulators and soluble plasma proteins that normally control consumptive complement activation in the fluid phase. In addition, the soluble regulators are equipped with domains that specifically recognize polyanionic surface markers that are specific for host structures. Thereby, these soluble regulators intensify the complement regulation by the membrane-bound regulators. In particular, the basement membranes in the eye and the kidney, which are exposed to the blood stream at the fenestration of the endothelium, appear particularly vulnerable to AP attack (3). Because basement membranes lack plasma-membrane bound regulators, their only means to protect themselves from AP attack is to recruit soluble regulators *via* exposing polyanionic host surface markers that attract the soluble regulators.

**Figure 1:**
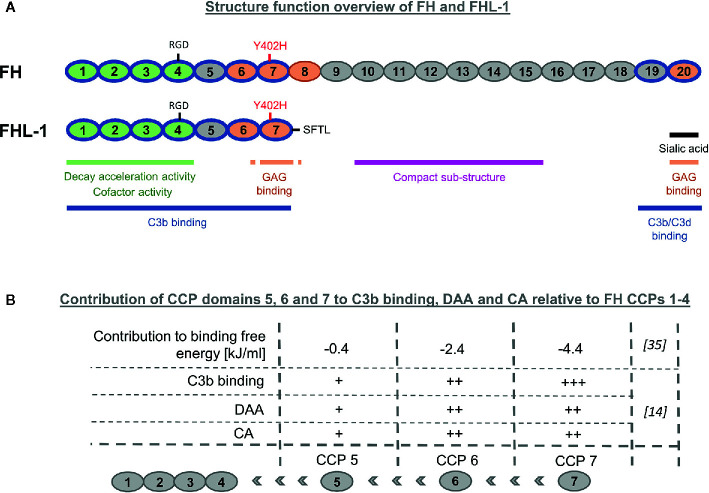
Structure-function overview of Factor H-like-1. **(A)** Comparison of FHL-1 and FH. FH and its splice product FHL-1 share the amino acid sequence and functionalities of the first seven N-terminal complement control protein (CCP) domains. FHL-1 lacks important host recognition properties which are located in the C-terminal CCP domains 19–20 of FH. **(B)** Impact of individual CCP domains 5, 6 and 7 to CCP 1–4 for binding on C3b, decay accelerating activity (DAA), and cofactor-activity (CA).

Factor H (FH) and Factor H-like 1 (FHL-1) are the only known negative fluid-phase regulators of the AP. FHL-1 was discovered by Schwaeble et al. (4) as a short transcript that was constantly expressed in the human liver and secreted into the blood stream. Because of the similarity to the FH N-terminus, it was assumed from the beginning that FHL-1 should share regulatory functionalities with FH. Later, it was clarified that this truncated form of FH with a molecular weight of 49 kDa originates from the *FH* gene by alternative splicing (5–7). The FH gene is located on chromosome 1q32 and is part of the Regulation of Complement Activation gene cluster (8, 9). FHL-1 is composed of the first seven N-terminal complement control protein (CCP) domains of FH but lacks the remaining 13 CCP domains (**Figure 1**). At its C-terminus, FHL-1 contains four unique amino acids (SFTL), which are derived from the alternative splicing and are encoded on exon 10 (6, 7). Remarkably, in mice, no evidence for an alternative FH splice variant was found (10).

With the discovery of the splice variant FHL-1 in humans, several questions arose as to the physiological relevance of this truncated FH version, the possible existence of new functional features in FHL-1, or the functional consequences on AP regulation introduced by the deletion of the 13 C-terminal FH domains (4, 11). However, to date, the physiological role of FHL-1 remains the subject of controversial discussion. This review considers the possible physiological origin of this molecule and summarizes the current knowledge of its regulatory activities as well as its physiological and pathophysiological roles.

## Molecular Insights Into the Regulatory Activity of FHL-1 in Comparison to FH

FH is known to display several functional properties, which are: binding C3b, competition with Factor B for C3b binding, co-factor activity (CA) for Factor I-mediated cleavage of C3b, decay accelerating activity (DAA) for the C3 convertase C3bBb, and host recognition through binding to specific polyanionic markers on the surface of host cells. C3b binding is a precondition for all complement regulatory activities of FH. Binding to C3 activation fragments was initially described with the discovery of FHL-1 and later assessed again by testing binding properties of recombinant FHL-1 and several fragments thereof to C3(H_2_O) (4, 12). CCP1–4 were sufficient for C3(H_2_O) binding, while further addition of the CCP domains 5, 6, and 7 increased the binding strength. This is in agreement with recent studies using surface plasmon resonance (SPR) for quantification of the binding affinity to C3b. The addition of the remaining CCP domains 5, 6, and 7 to CCP1–4 increased the affinity for C3b approximately 15 times, with an equilibrium dissociation constant of FHL-1 for C3b in the low micromolar range (~1 µM for FHL-1) (13, 14). By comparison, full-length FH displayed higher affinity for C3b (~0.6 µM), which is unsurprising, given that FH contains a second C3b binding patch within its two C-terminal domains 19–20 (15, 16). Some studies report a weak, third binding site for C3b located towards the middle of FH, while others could not detect it (15, 17). The difference between the binding affinity of FH and FHL-1 for C3b (and thus two *versus* one strong C3b-binding patch) appears less than expected. This may be explained by a complex structural model of FH, in which the C3b binding site in the C-terminal CCP domains is blocked or shielded by the N-terminal domains and only becomes accessible upon binding to polyanionic host surfaces marker. Several lines of evidence support the notion of such a structural FH model (13, 18–22). Through an absence of CCP domains 19–20, FHL-1 loses not only the second strong C3b binding site, but also the most critical host recognition site located in CCP20, which enables specific binding to glycosaminoglycans (GAG) and certain sialic acid-linked moieties (23–25). Therefore, the specificity for AP regulation on host surfaces for the splice variant FHL-1 could only derive from the GAG binding site located in CCP domain 7 (15, 26–29). On host-like sheep erythrocytes only FH CCPs 19–20 (carrying sialic acid and GAG binding properties), but not FH CCPs 6–8 (entailing only GAG binding functionality) exhibited functional competition with FH (24). A direct analysis of FH and FHL-1 regulatory capacity and surface specificity (host *versus* foreign) in FH/FHL-1-depleted serum revealed that both regulators protect foreign cell surfaces from complement attack at high concentrations, but FH was significantly more efficient in differentiating between foreign and host surfaces known to contain sialic acid moieties (14). This is in agreement with previous reports showing increased FH binding to sialylated surfaces (30, 31). These results show that the sialic acid (and GAG) binding site in CCP20 is much more important for self *versus* non-self discrimination than the GAG binding site in CCP7.

Misasi et al. (32) isolated FHL-1 from human plasma and demonstrated that it indeed harbors CA for the cleavage of C3b to iC3b (32). The precise determination of the involved CCP domains was demonstrated with experiments using FHL-1 and truncated fragments, with the result that the minimal functional unit to contain CA was CCP1–4 (33, 34). Similar to C3b binding, the successive addition of CCP domains to CCP1–4 enhanced the activity, while the addition of CCP6 to CCP1–5 had the largest impact on overall regulatory function (14, 35, 36). However, already in the fluid phase, when sialic acid binding does not even have a role in direct comparison to FH, FHL-1 appears to be a significantly weaker cofactor for Factor I-mediated C3b degradation (13, 14). Remarkably, this is also observed for the engineered versions of FH like miniFH. Although miniFH contains the N-terminal four and C-terminal two CCP domains of FH and thus exhibits a similar affinity for C3b as FH, miniFH is also a substantially weaker cofactor than FH (13, 14). This indicates that C3b affinity does not translate proportionally to CA. The structural complex of C3b with miniFH and Factor I revealed that only the two FH CCPs 2 and 3 directly contact Factor I (37). Processing of C3b by Factor I only occurs in ternary complexes of C3b with Factor I and a cofactor. Speculatively, FH with its two C3b binding patches is able to form a more stable ternary complex with C3b and Factor I than can FHL-1 or miniFH. This may be particularly true for the second cleavage of iC3b_1_ into iC3b_2_, because it is envisaged that the CUB (C1r/C1s–Uegf–Bmp1) domain of C3b begins to become partially unfolded upon consecutive Factor I cleavages (38).

Regarding the DAA of FHL-1, the literature is contradictory. FHL-1 was shown to act approximately 100 times weaker as a decay accelerator in comparison with FH when sheep erythrocytes deposited with C3 convertases were exposed to FH, FHL-1 or N-terminal fragments of FHL-1 (12). Nevertheless, it was shown unequivocally that FH CCPs 1–4 are the minimal requirement for fulfilling DAA. A recent SPR-based study, in which C3 convertases were formed on-chip, investigated the ability of FH, FHL-1 and FH-fragments to cause the decay of convertases. The outcome does not mirror the previous finding, because FHL-1 exhibited comparable DAA as full-length FH (14). The different outcomes could be attributed to differences in the assay. In the first report, the assay was performed on convertase pre-coated cells rather than in more defined (but less physiological) conditions on the SPR chip surface. However, because the solubility of FHL-1 in aqueous buffer at physiological pH and salt concentrations is rather limited, assays that require higher concentrations of FHL-1 stock solutions to be diluted into the assay conditions may need to be optimised for FHL-1 solubility (13, 14). In addition to specialized assays that investigate CA or DAA, recently the activities of FH and FHL-1 were directly compared in several serum assays. In these assays, the AP regulatory activities were nearly identical between the two protein variants when either FH or FHL-1 had been added to serum dilutions and consequently exposed to lipopolysaccharide-coated microtiter plates or complement vulnerable paroxysmal nocturnal haemoglobinuria cells (13). However, when FH and FHL-1 were compared on different self and non-self surfaces in FH/FHL-1-depleted serum, small difference in regulatory activity could be observed. FH was a slightly better regulator on self and self-like surfaces and a slightly worse regulator on foreign surfaces when compared to FHL-1. Additionally, although overall regulatory activity did not differ dramatically, it is obvious that FH does actively discriminate between self and non-self, whereas FHL-1 appears void of this capacity, at least for the surfaces tested (14).

To conclude, FHL-1 and FH share most of their complement regulatory features because they contain the first seven N-terminal CCP domains. FHL-1 lacks the important host recognition site at the FH C-terminus that recognises sialic acid structures [and does not contain another sialic acid binding moiety within its seven domains (24)], explaining the lack of differentiation between host and foreign surfaces tested. Overall, as evaluated in several serum assays, FHL-1 exhibits comparable activity to FH in protecting host cells (14).

## Physiological Role of FHL-1

Continuous low-level activation of the AP by probing all surfaces requires constant readiness for action of the solvent based negative regulators, FHL-1 and FH, to avoid wasteful consumption of the complement components in the fluid phase and self-directed damage on host surfaces. The regulators must satisfy two major requirements: displaying regulatory activity and being sufficiently abundant. Although FH is more selective and has slightly higher activity on host surfaces than FHL-1, both display overall comparable regulatory activities (discussed above). Therefore, the answer to the question as to which of the variants is the main AP regulator lies in the relative abundance of the two proteins. The determination of the plasma FHL-1 concentration proved to be difficult because to date there is no (commercially) available monoclonal antibody which specifically detects FHL-1 but not FH. FH was consistently reported to occur in the circulation at a concentration of ~2–3 µM (39–41), whereas the blood FHL-1 concentration varied in several reports over a large concentration range. Friese et al. used an indirect enzyme-linked immunosorbent assay-based subtraction method in which the FHL-1 concentration was determined by detecting either both, FH and FHL-1, or only FH with appropriate polyclonal antisera. This resulted in a FHL-1 serum concentration of ~1 µM (42). In a recent report, the FHL-1 plasma concentration was determined using western blot analysis (14). Briefly, for semi-quantitative comparison, standards were prepared adding purified FH/FHL-1 proteins into FH/FHL-1-depleted serum to achieve different defined concentrations. The strength of these “reference” FH/FHL-1 bands was then semi-quantitatively compared with FH/FHL-1 bands derived from different donor sera. This determination method delivered serum concentrations for FHL-1 of approximately 0.04 µM (14) (**Table 1**). Schwaeble et al. reported similar FH and FHL-1 mRNA levels in the liver, but plasma protein concentrations are also driven by clearance from the circulation (4). Indeed, pharmacokinetic analysis of human FH and FHL-1 applied intravenously into mice demonstrated the splice variant to be cleared much more rapidly from the murine circulation (14). The rapid plasma clearance of FHL-1 and its resulting low plasma concentration indicate that FH is the major systemic regulator of the AP, although the regulatory activities are not dramatically different, making it increasingly unlikely that FHL-1 plays a major role in systemic AP control (14). These observations may also argue that the physiological role of FHL-1 lies in the protection of specific tissues. Although located on the same gene and sharing the same promotor as well as transcription start site, both molecules displayed distinct expression patterns in some tissues and cell lines (42–45). In accordance with this, a different molecular FH/FHL-1 ratio was also observed in Bruch’s membrane, a layer of extracellular matrix positioned in the eye between the retinal pigment epithelium (RPE) and choroid blood vessels (46). FHL-1 was identified as the predominant AP regulator expressed by RPE cells. In contrast to FH, the splice variant is also able to diffuse from the choroid through the Bruch’s membrane. In addition to the higher expression levels by the RPE, FHL-1 also appears to be supplied to the eye from the systemic blood compartment *via* diffusion through the Bruch’s membrane, strongly indicating FHL-1 to be the major regulator at the RPE/Bruch’s membrane interface (46, 47). It is envisaged that FHL-1 can localize to certain host surface structures *via* its GAG binding site in CCP7, thus, preventing uncontrolled complement activation on such surfaces (8, 48). These findings on FHL-1 are particularly relevant because Bruch’s membrane is the site where drusen formation and tissue damage occur that are associated with the progressive eye disease age-related-macular degeneration (AMD)—the major cause of blindness in the older subjects in the western world (49). A polymorphism in CCP domain 7 (Y402H) is directly associated with an increased risk for developing AMD (50–53). Although the overall tertiary structure remains similar, the single nucleotide polymorphism (SNP) diminishes binding capability to heparan sulfate, which is the major GAG on Bruch’s membrane and, therefore, the major interaction site for FHL-1 (8, 27, 28, 46, 48, 54). Of note, the same Y402H SNP within full length FH displayed none or only a marginal impact on GAG binding, whereas when within FHL-1, dramatic differences occurred, which can be explained by two *versus* just one GAG binding site being present, respectively (27). The proven loss and desulfation of heparan sulfate GAGs on Bruch’s membrane with age and the resulting loss of binding sites for FHL-1 further supports the pathophysiological role of FHL-1, providing an explanation for the fact that particularly older people are affected (46, 55, 56).

**Table 1:** Comparison of FH and FHL-1.

Property	Protein		Reference
	FH	FHL-1	
Blood concentration [µM]	~2-3	0.04 (or up to ~1, see text)	(14, 40–43)
ß-phase plasma halftime of human proteins in mice [h]	18.3 ± 3.7	2.9 ± 0.5	(14)
*K*_D_ for C3b binding [µM]	~0.6	~1	(13, 14))
Cofactor activity (fluid phase)	++	+	(13, 14)
Decay accelerating activity (on SPR chip)	+	++	(13, 14)
Sialic acid binding (NMR saturation transfer technique)	+	none	(23, 24)
IC_50_ for PNH RBC protection [µM] (when added to FH/FHL-1-depleted serum)	1.4 ± 0.2	2.2 ± 0.5	(14)
IC_50_ for desialylated PNH RBC protection [µM] (when added to FH/FHL-1-depleted serum)	3.4 ± 0.8	2.2 ± 0.6	(14)
Deregulation by FHR-1	++	0 to +	(14)

## Pathophysiological Role of FHL-1

In the further course of investigating the pathophysiological role of the Y402H SNP in CCP 7, several further binding partners have been implicated, but it appears that no uniformly accepted conclusion has to date been reached. Functional consequences of the Y402H SNP have been described for, including, among others, C-reactive protein, pentraxin-3, oxidation end products, and zinc ions (57–60). Knowing the importance of FHL-1 on Bruch’s membrane, the question arose whether other membranes with a similar composition to that of Bruch’s membrane, like the kidney glomerular basement membrane, are also predominantly controlled by FHL-1. However, it was shown that the GAG binding site in CCP19–20 of FH is mainly involved in the interaction within the kidney, indicating that different GAG signatures can exist at different basement membranes, thus questioning a role for FHL-1 at the kidney basement membrane (61).

In contrast to proteoglycan layers, which constitute the basement membranes and heavily rely on the soluble complement regulators in plasma, human cell plasma membranes express a mix of membrane-bound complement regulators, including, for example, CR1 (CD35), MCP (CD46), DAF (CD55) and/or CD59, and hence the soluble complement regulators function ‘only’ as an important addition to the membrane tethered regulators on cellular surfaces (2, 62, 63). However, some tumor cell lines were reported to use primarily FH/FHL-1 for complement evasion. H2-glioblastoma cells were shown to upregulate FH and FHL-1 expression, with overall higher expression levels of the splice variant (64). Increased amounts of FHL-1 were also synthesized by the ovarian cell lines SK-OV-3 and Caov-3 and were detected in their direct microenvironment (65). Increased FHL-1 levels could increase local control of complement activation.

Soluble complement regulators are also attractive targets of pathogens that recruit regulators to their surface to evade the complement immune surveillance mechanism. Different bacteria, fungi, and parasites have been identified to specifically capture FH [reviewed in (66)]. Remarkably, some pathogens preferentially or even exclusively recruit FHL-1 although the amino acid sequence is identical to that of FH, except for the unique four amino acid patch at the FHL-1 C-terminus. One example is the M-protein of some group A *Streptococcus* strains, which was shown to enable binding to the CCP-7 domains of FH/FHL-1 with higher affinity for FHL-1 (11, 67, 68). Other pathogens that recruit or even preferentially recruit FHL-1 for immune evasion are *Plasmodium falciparum* and the spirochete *Borrelia spielmanii*, respectively (69, 70). Moreover, McDowell et al. identified a small surface protein-exposed on *Treponema denticola*, a bacterium involved in periodontal disease, which appears to preferentially bind FHL-1 (71, 72). However, they demonstrated an FI-independent cleavage of C3b and suggested that the purpose of FHL-1 being recruited by *Treponema denticola* is adherence to human cells rather than immune evasion. Such specific adhesive properties for FHL-1 were reported previously by Hellwage et al. (73). Both, FH and FHL-1 bear the amino acid sequence RGD in CCP domain 4, a sequence that is also found in adhesive proteins, including vitronectin and fibronectin (74). In contrast to FH, only FHL-1 could act (when coated on a ‘chamber slide’) as a matrix for adherence and spreading of the tested cell lines by binding to integrin receptors, potentially allowing effectors cells to bind via their integrin receptors to the RGD motif of FHL-1 bound to C3b-opsonized surfaces bridging the humoral and cellular immune responses (73).

## Discussion

Over time, the perception of FHL-1 has changed because several studies showed that the splice variant is a unique molecule with many shared but also some unique properties compared to FH. But even now, the benefit of producing a truncated form of FH is not completely clear. For some specialized tissues, FHL-1 could be a tailor-made fluid phase regulator with specialized properties, for example, being able to diffuse through certain basement membranes. The importance of FHL-1 as a tissue-specific regulator may be supported by its relatively late appearance in evolution, indicating a possible coevolution of this splice variant with a specialized tissue that relies on the protection of FHL-1. An *in-silico* gene analysis revealed that similar gene structures to FH that allow for alternative splicing of a truncated FH version are not found prior to the order of old-world monkeys (14). Another topic of ongoing research, which directly relates to specialized FHL-1 functions, touches on the deregulation functionality of Factor H-related proteins (FHR). Because it lacks the C-terminal CCP domains of FH, FHL-1 was thought to be less prone to deregulation by FH-related proteins than FH. Additionally, although some competition between FHR molecules and FHL-1 for selected functions have been observed (75), in AP serum assays on host cells, the deregulation by FHR-1 was considerably less for the splice variant FHL-1 than for FH (14). However, future studies are needed to further clarify the impact of deregulation of FH and its splice version by different FHRs. Future insights into how and why certain tissues modulate the splicing rates, and hence the relative expression levels of FH and FHL-1 will be fundamental in understanding the precise physiological role of FHL-1. As to why certain cancer types favor the relative expression of FHL-1 over FH, it can be speculated that by expressing FHL-1 an almost identical level of AP regulation can be achieved by using up much fewer resources, such that the energy for the production of 13 CCPs can be spared, which, however, comes at the expense of selectivity between self and non-self surfaces. To better define the role of FHL-1, it will also be important to identify further compartments that display altered FH/FHL-1 ratios compared to that in systemic circulation. To date, the synovial fluid expression levels of FH and FHL-1 in the settings of rheumatoid arthritis and the Bruch’s membrane have been determined (46, 76). Other interesting body fluids in specialized compartments (which may not require as stringent a selectivity for AP regulation as the systemic circulation and hence might benefit from higher FHL-1/FH ratios) might, for example, be the cerebrospinal and the pleural fluids. However, research of FHL-1 remains challenging. Its low solubility in phosphate-buffered saline complicates the daily handling of many standard laboratory assays (13, 14). The absence of FHL-1 in mice and thus the lack of the opportunity to investigate the functional consequences of engineered FHL-1 knock-out mice further complicate the characterization and importance of the FH splice variant FHL-1. However, the recent findings that FHL-1 is almost as active in down-regulating the AP as FH, albeit being less selective for host tissues, in conjunction with FHL-1 being selectively expressed at higher levels in certain tissues, underline the unique role for the splice variant FHL-1, which in the future is expected to be more intensely studied and thus be better understood.

## Author Contributions

All authors contributed to the article and approved the submitted version.

## Funding

This work was supported by the Deutsche Forschungsgemeinschaft grant (SCHM3018/2-2 to CS).

## Conflict of Interest

CS and MH-L are inventors of a patent application that describes the use of engineered complement regulatory proteins for therapeutic applications. They received honoraria for speaking at symposia organized by Alexion Pharmaceuticals.

The remaining authors declare that the research was conducted in the absence of any commercial or financial relationships that could be construed as a potential conflict of interest.

## References

[1] Ricklin D Hajishengallis G Yang K Lambris JD Complement: a key system for immune surveillance and homeostasis. Nat Immunol (2010) 11:785–97. 10.1038/ni.1923 PMC2924908 20720586

[2] Schmidt CQ Lambris JD Ricklin D Protection of host cells by complement regulators. Immunol Rev (2016) 274:152–71. 10.1111/imr.12475 PMC5432642 27782321

[3] Borza D-B Glomerular basement membrane heparan sulfate in health and disease: A regulator of local complement activation. Matrix Biol J Int Soc Matrix Biol (2017) 57–58:299–310. 10.1016/j.matbio.2016.09.002 PMC5026315 27609404

[4] Schwaeble W Zwirner J Schulz TF Linke RP Dierich MP Weiss EH Human complement factor H: expression of an additional truncated gene product of 43 kDa in human liver. Eur J Immunol (1987) 17:1485–9. 10.1002/eji.1830171015 2445583

[5] Estaller C Schwaeble W Dierich M Weiss EH Human complement factor H: two factor H proteins are derived from alternatively spliced transcripts. Eur J Immunol (1991) 21:799–802. 10.1002/eji.1830210337 1826264

[6] Sim RB Kölble K McAleer MA Dominguez O Dee VM Genetics and deficiencies of the soluble regulatory proteins of the complement system. Int Rev Immunol (1993) 10:65–86. 10.3109/08830189309051172 8340678

[7] Ripoche J Day AJ Harris TJ Sim RB The complete amino acid sequence of human complement factor H. Biochem J (1988) 249:593–602. 10.1042/bj2490593 2963625 PMC1148743

[8] Rodríguez de Córdoba S Esparza-Gordillo J Goicoechea de Jorge E Lopez-Trascasa M Sánchez-Corral P The human complement factor H: functional roles, genetic variations and disease associations. Mol Immunol (2004) 41:355–67. 10.1016/j.molimm.2004.02.005 15163532

[9] Rodriguez de Cordoba S Lublin DM Rubinstein P Atkinson JP Human genes for three complement components that regulate the activation of C3 are tightly linked. J Exp Med (1985) 161:1189–95. 10.1084/jem.161.5.1189 PMC2187593 3157763

[10] Vik DP Keeney JB Muñoz-Cánoves P Chaplin DD Tack BF Structure of the murine complement factor H gene. J Biol Chem (1988) 263:16720–4. 2972715

[11] Zipfel PF Skerka C FHL-1/reconectin: a human complement and immune regulator with cell-adhesive function. Immunol Today (1999) 20:135–40. 10.1016/s0167-5699(98)01432-7 10203705

[12] Kühn S Zipfel PF Mapping of the domains required for decay acceleration activity of the human factor H-like protein 1 and factor H. Eur J Immunol (1996) 26:2383–7. 10.1002/eji.1830261017 8898949

[13] Harder MJ Anliker M Höchsmann B Simmet T Huber-Lang M Schrezenmeier H Comparative Analysis of Novel Complement-Targeted Inhibitors, MiniFH, and the Natural Regulators Factor H and Factor H-like Protein 1 Reveal Functional Determinants of Complement Regulation. J Immunol Baltim Md 1950 (2016) 196:866–76. 10.4049/jimmunol.1501919 PMC4707092 26643478

[14] Dopler A Guntau L Harder MJ Palmer A Höchsmann B Schrezenmeier H Self versus Nonself Discrimination by the Soluble Complement Regulators Factor H and FHL-1. J Immunol Baltim Md 1950 (2019) 202:2082–94. 10.4049/jimmunol.1801545 30745459

[15] Schmidt CQ Herbert AP Kavanagh D Gandy C Fenton CJ Blaum BS A New Map of Glycosaminoglycan and C3b Binding Sites on Factor H. J Immunol (2008) 181:2610–9. 10.4049/jimmunol.181.4.2610 18684951

[16] Makou E Bailey RG Johnston H Parkin JD Hulme AN Hähner G Combining SPR with atomic-force microscopy enables single-molecule insights into activation and suppression of the complement cascade. J Biol Chem (2019) 294:20148–63. 10.1074/jbc.RA119.010913 PMC6937562 31719147

[17] Haque A Cortes C Alam MN Sreedhar M Ferreira VP Pangburn MK Characterization of Binding Properties of Individual Functional Sites of Human Complement Factor H. Front Immunol (2020) 11:1728:1728. 10.3389/fimmu.2020.01728 32849614 PMC7417313

[18] Aslam M Perkins SJ Folded-back solution structure of monomeric factor H of human complement by synchrotron X-ray and neutron scattering, analytical ultracentrifugation and constrained molecular modelling. J Mol Biol (2001) 309:1117–38. 10.1006/jmbi.2001.4720 11399083

[19] Oppermann M Manuelian T Józsi M Brandt E Jokiranta TS Heinen S The C-terminus of complement regulator Factor H mediates target recognition: evidence for a compact conformation of the native protein. Clin Exp Immunol (2006) 144:342–52. 10.1111/j.1365-2249.2006.03071.x PMC1809651 16634809

[20] Józsi M Oppermann M Lambris JD Zipfel PF The C-terminus of complement factor H is essential for host cell protection. Mol Immunol (2007) 44:2697–706. 10.1016/j.molimm.2006.12.001 PMC2700862 17208302

[21] Herbert AP Makou E Chen ZA Kerr H Richards A Rappsilber J Complement Evasion Mediated by Enhancement of Captured Factor H: Implications for Protection of Self-Surfaces from Complement. J Immunol Baltim Md 1950 (2015) 195:4986–98. 10.4049/jimmunol.1501388 PMC4635569 26459349

[22] Pouw RB Brouwer MC de Gast M van Beek AE van den Heuvel LP Schmidt CQ Potentiation of complement regulator factor H protects human endothelial cells from complement attack in aHUS sera. Blood Adv (2019) 3:621–32. 10.1182/bloodadvances.2018025692 PMC6391659 30804016

[23] Blaum BS Hannan JP Herbert AP Kavanagh D Uhrín D Stehle T Structural basis for sialic acid-mediated self-recognition by complement factor H. Nat Chem Biol (2015) 11:77–82. 10.1038/nchembio.1696 25402769

[24] Schmidt CQ Hipgrave Ederveen AL Harder MJ Wuhrer M Stehle T Blaum BS Biophysical analysis of sialic acid recognition by the complement regulator Factor H. Glycobiology (2018) 28:765–73. 10.1093/glycob/cwy061 PMC6142864 29982679

[25] Ferreira VP Herbert AP Hocking HG Barlow PN Pangburn MK Critical role of the C-terminal domains of factor H in regulating complement activation at cell surfaces. J Immunol Baltim Md 1950 (2006) 177:6308–16. 10.4049/jimmunol.177.9.6308 17056561

[26] Blackmore TK Sadlon TA Ward HM Lublin DM Gordon DL Identification of a heparin binding domain in the seventh short consensus repeat of complement factor H. J Immunol Baltim Md 1950 (1996) 157:5422–7. 8955190

[27] Herbert AP Uhrín D Lyon M Pangburn MK Barlow PN Disease-associated sequence variations congregate in a polyanion recognition patch on human factor H revealed in three-dimensional structure. J Biol Chem (2006) 281:16512–20. 10.1074/jbc.M513611200 16533809

[28] Prosser BE Johnson S Roversi P Herbert AP Blaum BS Tyrrell J Structural basis for complement factor H linked age-related macular degeneration. J Exp Med (2007) 204:2277–83. 10.1084/jem.20071069 PMC2118454 17893204

[29] Schmidt CQ Herbert AP Hocking HG Uhrín D Barlow PN Translational mini-review series on complement factor H: structural and functional correlations for factor H. Clin Exp Immunol (2008) 151:14–24. 10.1111/j.1365-2249.2007.03553.x 18081691 PMC2276926

[30] Kazatchkine MD Fearon DT Silbert JE Austen KF Surface-associated heparin inhibits zymosan-induced activation of the human alternative complement pathway by augmenting the regulatory action of the control proteins on particle-bound C3b. J Exp Med (1979) 150:1202–15. 10.1084/jem.150.5.1202 PMC2185702 501288

[31] Meri S Pangburn MK Discrimination between activators and nonactivators of the alternative pathway of complement: regulation via a sialic acid/polyanion binding site on factor H. Proc Natl Acad Sci U S A (1990) 87:3982–6. 10.1073/pnas.87.10.3982 PMC54028 1692629

[32] Misasi R Huemer HP Schwaeble W Sölder E Larcher C Dierich MP Human complement factor H: an additional gene product of 43 kDa isolated from human plasma shows cofactor activity for the cleavage of the third component of complement. Eur J Immunol (1989) 19:1765–8. 10.1002/eji.1830190936 2529127

[33] Kühn S Skerka C Zipfel PF Mapping of the complement regulatory domains in the human factor H-like protein 1 and in factor H1. J Immunol Baltim Md 1950 (1995) 155:5663–70. 7499851

[34] Gordon DL Kaufman RM Blackmore TK Kwong J Lublin DM Identification of complement regulatory domains in human factor H. J Immunol Baltim Md 1950 (1995) 155:348–56. 7541419

[35] Kang J Warren AN Comment on “Self versus Nonself Discrimination by the Soluble Complement Regulators Factor H and FHL-1.” J Immunol Baltim Md 1950 (2019) 203:2029. 10.4049/jimmunol.1900660 31591257

[36] Dopler A Guntau L Harder MJ Palmer A Höchsmann B Schrezenmeier H Response to Comment on “Self versus Nonself Discrimination by the Soluble Complement Regulators Factor H and FHL-1.” J Immunol Baltim Md 1950 (2019) 203:2029–30. 10.4049/jimmunol.1900994 31591258

[37] Xue X Wu J Ricklin D Forneris F Di Crescenzio P Schmidt CQ Regulator-dependent mechanisms of C3b processing by factor I allow differentiation of immune responses. Nat Struct Mol Biol (2017) 24:643–51. 10.1038/nsmb.3427 PMC5773341 28671664

[38] Sahu A Isaacs SN Soulika AM Lambris JD Interaction of vaccinia virus complement control protein with human complement proteins: factor I-mediated degradation of C3b to iC3b1 inactivates the alternative complement pathway. J Immunol Baltim Md 1950 (1998) 160:5596–604. 9605165

[39] Weiler JM Daha MR Austen KF Fearon DT Control of the amplification convertase of complement by the plasma protein beta1H. Proc Natl Acad Sci U S A (1976) 73:3268–72. 10.1073/pnas.73.9.3268 PMC431003 1067618

[40] Sofat R Mangione PP Gallimore JR Hakobyan S Hughes TR Shah T Distribution and determinants of circulating complement factor H concentration determined by a high-throughput immunonephelometric assay. J Immunol Methods (2013) 390:63–73. 10.1016/j.jim.2013.01.009 23376722

[41] Hakobyan S Harris CL Tortajada A Goicochea de Jorge E García-Layana A Fernández-Robredo P Measurement of factor H variants in plasma using variant-specific monoclonal antibodies: application to assessing risk of age-related macular degeneration. Invest Ophthalmol Vis Sci (2008) 49:1983–90. 10.1167/iovs.07-1523 18436830

[42] Friese MA Hellwage J Jokiranta TS Meri S Müller-Quernheim HJ Peter HH Different regulation of factor H and FHL-1/reconectin by inflammatory mediators and expression of the two proteins in rheumatoid arthritis (RA). Clin Exp Immunol (2000) 121:406–15. 10.1046/j.1365-2249.2000.01285.x PMC1905714 10931160

[43] Friese MA Hellwage J Jokiranta TS Meri S Peter HH Eibel H FHL-1/reconectin and factor H: two human complement regulators which are encoded by the same gene are differently expressed and regulated. Mol Immunol (1999) 36:809–18. 10.1016/s0161-5890(99)00101-7 10698334

[44] Morgan BP Daniels RH Williams BD Measurement of terminal complement complexes in rheumatoid arthritis. Clin Exp Immunol (1988) 73:473–8. PMC1541752 3208454

[45] Brodeur JP Ruddy S Schwartz LB Moxley G Synovial fluid levels of complement SC5b-9 and fragment Bb are elevated in patients with rheumatoid arthritis. Arthritis Rheum (1991) 34:1531–7. 10.1002/art.1780341209 1747138

[46] Clark SJ Schmidt CQ White AM Hakobyan S Morgan BP Bishop PN Identification of factor H-like protein 1 as the predominant complement regulator in Bruch’s membrane: implications for age-related macular degeneration. J Immunol Baltim Md 1950 (2014) 193:4962–70. 10.4049/jimmunol.1401613 PMC4225158 25305316

[47] Taylor RL Poulter JA Downes SM McKibbin M Khan KN Inglehearn CF Loss-of-Function Mutations in the CFH Gene Affecting Alternatively Encoded Factor H-like 1 Protein Cause Dominant Early-Onset Macular Drusen. Ophthalmology (2019) 126:1410–21. 10.1016/j.ophtha.2019.03.013 PMC6856713 30905644

[48] Clark SJ Higman VA Mulloy B Perkins SJ Lea SM Sim RB His-384 allotypic variant of factor H associated with age-related macular degeneration has different heparin binding properties from the non-disease-associated form. J Biol Chem (2006) 281:24713–20. 10.1074/jbc.M605083200 16787919

[49] Friedman DS O’Colmain BJ Muñoz B Tomany SC McCarty C de Jong PTVM Eye Diseases Prevalence Research Group. Prevalence of age-related macular degeneration in the United States. Arch Ophthalmol Chic Ill 1960 (2004) 122:564–72. 10.1001/archopht.122.4.564 15078675

[50] Hageman GS Anderson DH Johnson LV Hancox LS Taiber AJ Hardisty LI A common haplotype in the complement regulatory gene factor H (HF1/CFH) predisposes individuals to age-related macular degeneration. Proc Natl Acad Sci U S A (2005) 102:7227–32. 10.1073/pnas.0501536102 PMC1088171 15870199

[51] Haines JL Hauser MA Schmidt S Scott WK Olson LM Gallins P Complement factor H variant increases the risk of age-related macular degeneration. Science (2005) 308:419–21. 10.1126/science.1110359 15761120

[52] Klein RJ Zeiss C Chew EY Tsai J-Y Sackler RS Haynes C Complement factor H polymorphism in age-related macular degeneration. Science (2005) 308:385–9. 10.1126/science.1109557 PMC1512523 15761122

[53] Edwards AO Ritter R Abel KJ Manning A Panhuysen C Farrer LA Complement factor H polymorphism and age-related macular degeneration. Science (2005) 308:421–4. 10.1126/science.1110189 15761121

[54] Giannakis E Jokiranta TS Male DA Ranganathan S Ormsby RJ Fischetti VA A common site within factor H SCR 7 responsible for binding heparin, C-reactive protein and streptococcal M protein. Eur J Immunol (2003) 33:962–9. 10.1002/eji.200323541 12672062

[55] Keenan TDL Pickford CE Holley RJ Clark SJ Lin W Dowsey AW Age-dependent changes in heparan sulfate in human Bruch’s membrane: implications for age-related macular degeneration. Invest Ophthalmol Vis Sci (2014) 55:5370–9. 10.1167/iovs.14-14126 25074778

[56] Clark SJ Bishop PN Role of Factor H and Related Proteins in Regulating Complement Activation in the Macula, and Relevance to Age-Related Macular Degeneration. J Clin Med (2015) 4:18–31. 10.3390/jcm4010018 25729613 PMC4340553

[57] Swinkels M Zhang JH Tilakaratna V Black G Perveen R McHarg S C-reactive protein and pentraxin-3 binding of factor H-like protein 1 differs from complement factor H: implications for retinal inflammation. Sci Rep (2018) 8:1643. 10.1038/s41598-017-18395-7 29374201 PMC5786067

[58] Bhutto IA Baba T Merges C Juriasinghani V McLeod DS Lutty GA C-reactive protein and complement factor H in aged human eyes and eyes with age-related macular degeneration. Br J Ophthalmol (2011) 95:1323–30. 10.1136/bjo.2010.199216 PMC4916773 21633121

[59] Weismann D Hartvigsen K Lauer N Bennett KL Scholl HPN Charbel Issa P Complement factor H binds malondialdehyde epitopes and protects from oxidative stress. Nature (2011) 478:76–81. 10.1038/nature10449 21979047 PMC4826616

[60] Nan R Farabella I Schumacher FF Miller A Gor J Martin ACR Zinc binding to the Tyr402 and His402 allotypes of complement factor H: possible implications for age-related macular degeneration. J Mol Biol (2011) 408:714–35. 10.1016/j.jmb.2011.03.006 PMC3092982 21396937

[61] Clark SJ Ridge LA Herbert AP Hakobyan S Mulloy B Lennon R Tissue-specific host recognition by complement factor H is mediated by differential activities of its glycosaminoglycan-binding regions. J Immunol Baltim Md 1950 (2013) 190:2049–57. 10.4049/jimmunol.1201751 PMC3672945 23365078

[62] Morgan BP Meri S Membrane proteins that protect against complement lysis. Springer Semin Immunopathol (1994) 15:369–96. 10.1007/bf01837366 8153873

[63] Ferreira VP Pangburn MK Factor H mediated cell surface protection from complement is critical for the survival of PNH erythrocytes. Blood (2007) 110:2190–2. 10.1182/blood-2007-04-083170 PMC1976366 17554058

[64] Junnikkala S Jokiranta TS Friese MA Jarva H Zipfel PF Meri S Exceptional resistance of human H2 glioblastoma cells to complement-mediated killing by expression and utilization of factor H and factor H-like protein 1. J Immunol Baltim Md 1950 (2000) 164:6075–81. 10.4049/jimmunol.164.11.6075 10820293

[65] Junnikkala S Hakulinen J Jarva H Manuelian T Bjørge L Bützow R Secretion of soluble complement inhibitors factor H and factor H-like protein (FHL-1) by ovarian tumour cells. Br J Cancer (2002) 87:1119–27. 10.1038/sj.bjc.6600614 PMC2376183 12402151

[66] Ermert D Ram S Laabei M The hijackers guide to escaping complement: Lessons learned from pathogens. Mol Immunol (2019) 114:49–61. 10.1016/j.molimm.2019.07.018 31336249

[67] Peterson PK Schmeling D Cleary PP Wilkinson BJ Kim Y Quie PG Inhibition of alternative complement pathway opsonization by group A streptococcal M protein. J Infect Dis (1979) 139:575–85. 10.1093/infdis/139.5.575 374652

[68] Kotarsky H Hellwage J Johnsson E Skerka C Svensson HG Lindahl G Identification of a domain in human factor H and factor H-like protein-1 required for the interaction with streptococcal M proteins. J Immunol Baltim Md 1950 (1998) 160:3349–54. 9531294

[69] Herzberger P Siegel C Skerka C Fingerle V Schulte-Spechtel U Wilske B Identification and characterization of the factor H and FHL-1 binding complement regulator-acquiring surface protein 1 of the Lyme disease spirochete Borrelia spielmanii sp. nov. Int J Med Microbiol IJMM (2009) 299:141–54. 10.1016/j.ijmm.2008.06.005 18706858

[70] Kennedy AT Schmidt CQ Thompson JK Weiss GE Taechalertpaisarn T Gilson PR Recruitment of Factor H as a Novel Complement Evasion Strategy for Blood-Stage Plasmodium falciparum Infection. J Immunol Baltim Md 1950 (2016) 196:1239–48. 10.4049/jimmunol.1501581 26700768

[71] Simonson LG Goodman CH Bial JJ Morton HE Quantitative relationship of Treponema denticola to severity of periodontal disease. Infect Immun (1988) 56:726–8. 10.1128/IAI.56.4.726-728.1988 PMC259361 3346072

[72] McDowell JV Lankford J Stamm L Sadlon T Gordon DL Marconi RT Demonstration of factor H-like protein 1 binding to Treponema denticola, a pathogen associated with periodontal disease in humans. Infect Immun (2005) 73:7126–32. 10.1128/IAI.73.11.7126-7132.2005 PMC1273895 16239506

[73] Hellwage J Kühn S Zipfel PF The human complement regulatory factor-H-like protein 1, which represents a truncated form of factor H, displays cell-attachment activity. Biochem J (1997) 326:321–7. 10.1042/bj3260321 PMC1218672 9291099

[74] Hynes RO Integrins: versatility, modulation, and signaling in cell adhesion. Cell (1992) 69:11–25. 10.1016/0092-8674(92)90115-s 1555235

[75] Cipriani V Lorés-Motta L He F Fathalla D Tilakaratna V McHarg S Increased circulating levels of Factor H-Related Protein 4 are strongly associated with age-related macular degeneration. Nat Commun (2020) 11:778. 10.1038/s41467-020-14499-3 32034129 PMC7005798

[76] Friese MA Manuelian T Junnikkala S Hellwage J Meri S Peter HH Release of endogenous anti-inflammatory complement regulators FHL-1 and factor H protects synovial fibroblasts during rheumatoid arthritis. Clin Exp Immunol (2003) 132:485–95. 10.1046/j.1365-2249.2003.02173.x PMC1808733 12780697

